# Mechanistic and biophysical characterization of polymyxin resistance response regulator PmrA in *Acinetobacter baumannii*

**DOI:** 10.3389/fmicb.2024.1293990

**Published:** 2024-02-27

**Authors:** Zhenlin Ouyang, Wenbo He, Min Jiao, Qinyue Yu, Yucheng Guo, Moath Refat, Qian Qin, Jiaxin Zhang, Qindong Shi, Fang Zheng, Yurong Wen

**Affiliations:** ^1^Shaanxi Provincial Key Laboratory of Sepsis in Critical Care Medicine, Department of Critical Care Medicine, Center for Microbiome Research of Med-X Institute, The First Affiliated Hospital, Xi’an Jiaotong University, Xi’an, China; ^2^The Key Laboratory of Environment and Genes Related to Disease of Ministry of Education Health Science Center, Xi’an Jiaotong University, Xi’an, China

**Keywords:** polymyxin resistance, response regulator, *Acinetobacter baumannii*, PmrA, mechanism

## Abstract

**Introduction:**

*Acinetobacter baumannii* PmrAB is a crucial two-component regulatory system (TCS) that plays a vital role in conferring resistance to polymyxin. PmrA, a response regulator belonging to the OmpR/PhoB family, is composed of a C-terminal DNA-binding effector domain and an N-terminal receiver domain. The receiver domain can be phosphorylated by PmrB, a transmembrane sensor histidine kinase that interacts with PmrA. Once phosphorylated, PmrA undergoes a conformational change, resulting in the formation of a symmetric dimer in the receiver domain. This conformational change facilitates the recognition of promoter DNA by the DNA-binding domain of PmrA, leading to the activation of adaptive responses.

**Methods:**

X-ray crystallography was carried out to solve the structure of PmrA receiver domain. Electrophoretic mobility shift assay and Isothermal titration calorimetry were recruited to validate the interaction between the recombinant PmrA protein and target DNA. Field-emission scanning electron microscopy (FE-SEM) was employed to characterize the surface morphology of *A. baumannii* in both the PmrA knockout and mutation strains.

**Results:**

The receiver domain of PmrA follows the canonical α5β5 response regulator assembly, which undergoes dimerization upon phosphorylation and activation. Beryllium trifluoride is utilized as an aspartate phosphorylation mimic in this process. Mutations involved in phosphorylation and dimerization significantly affected the expression of downstream *pmrC* and *naxD* genes. This impact resulted in an enhanced cell surface smoothness with fewer modifications, ultimately contributing to a decrease in colistin (polymyxin E) and polymyxin B resistance. Additionally, a conservative direct-repeat DNA PmrA binding sequence TTTAAGNNNNNTTTAAG was identified at the promoter region of the *pmrC* and *naxD* gene. These findings provide structural insights into the PmrA receiver domain and reveal the mechanism of polymyxin resistance, suggesting that PmrA could be a potential drug target to reverse polymyxin resistance in *Acinetobacter baumannii*.

## 1 Introduction

The emergence of multidrug-resistant *Acinetobacter baumannii* infection poses a significant threat as an opportunistic and medically important pathogen, causing a wide range of nosocomial infections (Almasaudi, 2018). This bacterium exhibits a remarkable ability to acquire resistance to multiple antibiotics, with up to 30% of clinical isolates of *A. baumannii* demonstrating resistance to at least three antibiotics commonly used in intensive care units (Uc-Cachón et al., 2019). Antimicrobial-resistant *A. baumannii* strains, including commonly used clinical antibiotics such as imipenem, sulbactam, rifampin, and tigecycline resistant strains, have been isolated with increasing frequency. The situation has led to a rise in the use of polymyxins as antimicrobial agents, resulting in the emergence of strains resistant to polymyxins (Hamidian and Nigro, 2019; Hashemi et al., 2019). *A. baumannii* strains are resistant to a wide range of currently available antibiotics and are of particular concern, due to their potential for spreading within critical care environments (Büchler et al., 2018; Lee et al., 2020). *A. baumannii* has been ranked as the most critical threat among multidrug-resistant bacteria by the World Health Organization due to its remarkable ability to develop resistance to multiple drugs (Bagińska et al., 2019).

Colistin (Polymyxin E) and polymyxin B are cyclic cationic peptides synthesized by the *Bacillus* species. Polymyxin E exhibits a rapid bactericidal effect against Gram-negative bacteria by interacting with the lipid A moiety of lipopolysaccharide (LPS), which leads to the disorganization of the outer membrane (Vaara, 2009). According to reports, there are three main pathways that contribute to the development of polymyxin resistance: (1) specific modification of the lipid A component of the outer membrane lipopolysaccharide, resulting in a reduction of the net negative charge of the outer membrane. (2) Proteolytic cleavage of the antibiotic molecule. (3) Activation of a broad-spectrum efflux pump, which can expel a wide range of antibiotics from the bacterial cell (Duperthuy, 2020).

Lately, some questions have been raised regarding the underlying causes of polymyxin resistance. To date, the following three mechanisms have been firmly confirmed in *A. baumannii*: (1) The emergence of high levels of polymyxin resistance can occur when there is a complete loss of lipid A, leading to a deficient lipopolysaccharide (LPS) in the outer membrane. This resistance is attributed to spontaneous point mutations in genes involved in lipid A synthesis, namely lpxA, lpxC, or lpxD. (2) Acquired polymyxin resistance is commonly mediated by the replacement of lipid A with phosphoethanolamine (pEtN), a process regulated by the *pmrCAB* operon (Beceiro et al., 2011). The PmrAB TCS controls the expression of PmrC, which encodes the lipid A phosphoethanolamine transferase enzyme. This enzyme plays a crucial role in facilitating the addition of phosphoethanolamine (pEtN) to lipid A. Eliminating negative charges in this alteration impacts the interaction between polymyxin and the cell membrane (Samantha and Vrielink, 2020). (3) The alteration of galactosamine (GalN) is associated with the emergence of polymyxin resistance (Pelletier et al., 2013). N-acetylhexosamine deacetylase (NaxD) is a key enzyme for the deacetylation of N-acetylgalactosamine via attaching with the lipid carrier undecaprenyl phosphate. This step is essential for the subsequent addition of GalN galactosamine to lipid A (Gerson et al., 2020). Upon sensing of environmental stimuli, the PmrAB TCS subsequently facilitates *naxD* transcription (Chin et al., 2015). Furthermore, the development of polymyxin resistance has been attributed to the activation of a broad-spectrum efflux pump due to a mutation in *emrB* gene or alterations in membrane permeability (Lima et al., 2018; Uppalapati et al., 2020).

The response regulator PmrA (Uniprot No: E2FGC2) consists of two main domains: the N-terminal receiver domain (RD, residues 1–116), which responds to the phosphorylation signal from its cognate histidine kinase PmrB, and the C-terminal OmpR/PhoB type DNA binding domain (DBD, residues 129–223), which modulates the expression of the downstream target gene (Gao et al., 2019).

In this particular study, we focused on investigating the role of the PmrAB TCS in polymyxin resistance of *A. baumannii* and providing a deeper understanding of the field. Biophysical, structural biology, and related mutagenesis approaches were adopted to investigate how PmrAs recognize specific DNA sequences and regulate DNA reorganization through their receiver domain. We determined the crystal structure of the *A. baumannii* PmrA receiver domain at 1.6 Å resolution, significant insights at the molecular level, with details underlying the assembly of PmrA. We confirmed that PmrA activates transcription by binding to a conserved DNA motif within the promoters of two operons, namely *pmrC* and *naxD* genes. Additionally, PmrA activates transcription of the two lipA modification enzymes, *pmrC* and *naxD* operons, which play crucial roles in polymyxin resistance. Overall, the present study shed new insight into the regulatory mechanism of polymyxin resistance in *A. baumannii* by utilizing multidisciplinary techniques and providing enhanced comprehension of the field. The study may lead to potential strategies to develop antimicrobial drugs targeting polymyxin resistance pathway and benefit the clinical infection treatment associated with multidrug resistant *A. baumannii*.

## 2 Experimental methods

### 2.1 Bacterial strains, plasmids, growth conditions, and antibiotics

The bacterial strains and plasmids utilized in this study are summarized in Supplementary Table 2, while the primers employed for cloning and site mutagenesis are documented in Supplementary Table 3. Unless otherwise specified, all strains were cultivated in lysogeny broth (LB), which consists of 10 g/L tryptone (Oxoid, Waltham, MA, United States), 5 g/L yeast extract (Oxoid, Waltham, MA, United States), and 10 g/L NaCl (Sigma-Aldrich, Burlington, MA, United States). For selection purposes, the antibiotics tetracycline (Sigma-Aldrich, Burlington, MA, United States) (20 or 15 μg/mL) and kanamycin (Sigma-Aldrich, Burlington, MA, United States) (25 μg/mL) were added as required (Tucker et al., 2014).

### 2.2 Gene knockout construction uses REC_*AB*_ system

Chromosomal PmrA deletion was constructed using REC_*AB*_ system (Tucker et al., 2014). In brief, we harvested the 500 bp upstream and 500 bp downstream DNA fragments of the PmrA using BIO-RAD C1000 Touch PCR thermal cycler. These fragments were designed to flank the kanamycin resistance cassette, amplified from the pKD4 plasmid. *A. baumannii* ATCC19606 carrying pAT04 vector containing REC_*AB*_ was cultured in LB media supplemented with tetracycline (20 μg/mL). The bacteria were grown to mid-log phase (OD_600_ = 0.3–0.5) at 37°C supplemented with 2 mM IPTG, next bacteria were washed with ice-cold 10% glycerol three times. Approximately 5 μg PCR-product was electroporated into 100 uL *A. baumannii* ATCC19606, and kanamycin (25 μg/mL) was used to select cultivate mutant strains. The selected colonies were verified by PCR and DNA sequencing and then cure the plasmid pAT04 for further use.

For constructing IPTG-inducible PmrA complementation vectors, the primer sets listed in Table 1 were used to prepare the *pmrA* fragment by PCR. The pAT04-PmrA plasmid was generated through seamless cloning. The PCR based site-directed mutagenesis approach described before (Zheng et al., 2004) was used to construct point mutation. In summary, our methodology involved utilizing a PCR procedure that involves denaturing the DNA template, followed by annealing and extending mutagenic primers using a proprietary DNA polymerase. One distinctive aspect of this system is the removal of parental DNA using *Dpn?* endonuclease, which specifically digests methylated or hemimethylated sequences in the parental DNA. Next, the plasmids were electroporated into the ΔpmrA strains. The PmrA complementation and pointed mutation strains were validated by PCR and DNA sequencing.

**TABLE 1:** X-ray data collection and refinement statistics of PmrA RD.

Crystal	PmrA_Receiver domain
**Data collection**	
Spacegroup	P 1 21 1
a, b, c (Å)	32.1, 39.1, 92.2
α, β, γ (°)	90.0, 95.86, 90.0
Resolution (Å)	31.94–1.6 (1.66–1.6)
Rmeas	0.036 (0.419)
Multiplicity	3.1 (2.7)
CC (1/2)	0.999 (0.935)
I/σ(I)	18.56/2.56
Completeness (%)	99.18 (98.28)
Wilson B-factor (Å2)	23.05
**Refinement**	
Total reflections	95,955 (12,052)
Unique reflections	30,050 (2,921)
*R_*work*_/R_*free*_*	0.1890/0.2386
**Number of atoms**	
Protein	249
Water	161
Average B-factor (Å2)	32.79
Protein ADP (Å2)	32.22
Water ADP (Å2)	39.88
**Ramachandran plot**	
Favored/allowed (%)	98/2
**Root mean square deviation**	
Bond lengths (Å)	0.011
Bond angle (°)	1.18
PDB code	8IMW

Statistics for the highest-resolution shell are shown in parentheses.

### 2.3 Protein expression and purification

The full-length (PmrA FL, 1–244) and receiver domain (PmrA RD, 1–116) of *A. baumannii* PmrA were obtained through PCR and inserted into the pET28a vector with a 6× His tag at the N-terminus. The constructed plasmids were then transformed into *E. coli* BL21 (DE3) star host cells. The cells were grown in LB medium supplemented with kanamycin (100 μg/mL) at 37°C and 200 rpm until reaching mid-log phase (OD_600_ = 0.6–0.8). Following induction with 1 mM IPTG, the cultures were incubated for 8 h before harvesting the cells through centrifugation at 7,000 *g* (Beckman, Brea, CA, United States). The resulting cell pellets were frozen at −80°C for future use. The recombinant proteins were purified using Ni-NTA Superflow Cartridges (5 ml Qiagen) with ÄKTA pure™ chromatography system. The eluted protein fractions were concentrated and loaded onto a Superdex 75pg 16/60 size exclusion chromatography column (GE Healthcare, Grens, Switzerland). The column was pre-equilibrated with a standardized buffer solution containing 25 mM Tris (pH 8.0), 150 mM NaCl, and 5% glycerol. During the purification process, 5.3 mM BeSO_4_, 35 mM NaF, and 7 mM MgCl_2_ were added to facilitate the phosphorylation of PmrA in complex with BeF_3_^–^ and magnesium. The main protein fractions were further concentrated (> 98% purity as determined by mass spectrometry and SDS-PAGE). The protein concentration was measured using a NanoDrop^®^ spectrometer (Thermo Fisher, Waltham, MA, United States) with the extinction coefficient generated from the ExPASy ProtParam program^1^ (Gasteiger et al., 2005).

### 2.4 Crystallization and data collection

The recombinant protein of the PmrA receiver domain (PmrA RD) was concentrated to a concentration of 25 mg/mL for screening crystallization conditions. Crystallization screening was conducted at 20°C using a 392-well Hampton crystallization plate, employing commercial kits from Molecular Dimensions and Hampton Research. The sitting-drop vapor-diffusion method was employed for crystallization. The PmrA RD crystals were successfully obtained in a crystallization condition consisting of 0.1 M Sodium malonate at pH 6.0 and 12% w/v Polyethylene glycol 3,350. To prepare the crystals for data collection, all obtained crystals were immersed in a cryoprotectant solution composed of the reservoir solution supplemented with 30% glycerol. Subsequently, the crystals were cryo-cooled in liquid nitrogen to maintain their integrity during the data collection process. X-ray diffraction data were collected from a single crystal using a wavelength of 0.98 Å at the Shanghai Synchrotron Radiation Facility (SSRF) BL18U1 beamline. The data collection was performed at a temperature of 100K to minimize crystal movement and enhance data quality (Ouyang et al., 2021).

### 2.5 Structure determination and refinement

The X-ray diffraction data obtained were processed and scaled using XDS software (Kabsch, 2010). Detailed information and statistics regarding the data collection are summarized in Table 1. The structure of the PmrA RD protein was determined through molecular replacement, utilizing the *Staphylococcus aureus* ArlR receiver domain (PDB: 6IS3) as a search model (Ouyang et al., 2019), which shares a sequence identity of 41% with PmrA RD. Structural alignment and comparison, specifically the root mean square deviation (RMSD) calculation, were performed using PyMOL. The analysis of structure interfaces and assemblies was conducted using the PDBe PISA server (Krissinel and Henrick, 2007). The crystal structure of PmrA RD was ultimately solved at a resolution of 1.6 Å, belonging to space group P1211, with the following cell dimensions: *a* = 32.1 Å, *b* = 39.1 Å, *c* = 92.2 Å, and β = 95.9°, while α and γ angles were both 90°. COOT and PHENIX4 were used for model building and refinement (Emsley and Cowtan, 2004; Adams et al., 2010). Individual B-factor, non-crystallographic symmetry (NCS) torsion-angle, and translation/libration/screw (TLS) parameters were used in refinement strategies. The final structure was refined to a final Rwork/Rfree of 0.1890/0.2386, indicating the goodness-of-fit between the model and the experimental data.

### 2.6 Electrophoretic mobility shift assay

The electrophoretic mobility shift assay (EMSA) experiments were conducted according to the protocol provided by the LightShift Chemiluminescent EMSA Kit (Thermo Scientific, Waltham, MA, United States). A 200+ bp biotin-labeled DNA fragments from the upstream regions of *pmrC* (232 bp) and *naxD* (251 bp) were obtained by PCR using the 5′-end biotin-labeled primers listed in Supplementary Table 3. The mutated promoter DNA fragments, as control sequences, were obtained by Site directed mutagenesis PCR using primer listed in Supplementary Table 3. These fragments were used to detect interactions between proteins and DNA. The EMSA reaction followed a previously described protocol (Wen et al., 2017b). In brief, the binding reactions were incubated in a 10 μl volume, containing 1 pmol of DNA and various concentrations of PmrA FL protein, in the binding buffer (10 mM Tris, 50 mM KCl, 1 mM DTT, 5% glycerol, pH 7.5) for 30 min. The samples were then loaded directly onto a 5% native polyacrylamide gel and separated in 0.5× TBE buffer at 100 V on ice. Subsequently, the gels were transferred to a nylon membrane through electroblotting in 0.5× TBE buffer at 300 mA for 90 min at 4 °C. After cross-linking the DNA fragments at 120 mJ/cm^2^ using a commercial UV-light crosslinking instrument equipped with 254 nm bulbs, chemiluminescence was performed to detect the biotin-labeled DNA in the membranes.

### 2.7 Isothermal titration calorimetry

Isothermal titration calorimetry (ITC) experiments were all performed using a Microcal ITC200 calorimeter (GE Healthcare, Grens, Switzerland) at 25 °C. Before conducting the experiments, samples of DNA (Supplementary Table 3) and protein were prepared with the exact batch of buffer solution (25 mM Tris pH 8.0, 150 mM NaCl, 10% glycerol with or without BeF_3_^–^ and magnesium), using size-exclusion chromatography. During the purification process, 5.3 mM BeSO_4_, 35 mM NaF, and 7 mM MgCl_2_ were added for the phosphorylation of PmrA FL.

A volume of 60 μL solution containing PmrA FL protein (150 μM) was loaded into 15 μM of the 17 bp direct-repeat DNA to measure the enthalpy of protein to DNA association. The concentration of PmrA FL protein in the injection syringe ranged between 100 and 150 μM. Injections were performed with 0.4 μL for the first injection and 2 μL from the second to the nineteenth injection, with intervals of 120 s. The ITC data were analyzed using the Microcal ITC data analysis package, specifically utilizing the one-binding site mode, as part of the supplementary features (Wen et al., 2017a).

### 2.8 RNA isolation and real-time PCR

The PmrA knockout and site-directed mutagenesis strains were grown till reaching the mid-logarithmic phase in LB. The suspensions were then adjusted to an optical density of 0.01 at OD600 and grown for 5 h at 37°C and 200 rpm before harvesting. Total RNA isolation and cDNA synthesis were carried out following previously described methods (Ouyang et al., 2019). In brief, total RNA was extracted using a Qiagen RNeasy Mini kit. Subsequently, the High-Capacity cDNA Reverse Transcription Kit (Bio-Rad, Hercules, CA, United States) was used to synthesize first-strand cDNA from the total RNA, following the manufacturer’s instructions and employing 300 ng of total RNA as the template. Expression of *pmrC* and *naxD* were determined by two-step real-time PCR System (Bio-Rad, Hercules, CA, United States). Gene-specific primers were designed to generate specific products of 200 to 250 bp in size (Supplementary Table 3) and annealed at 60°C to account for any variations in the initial material amount. The 16S RNA gene was used as a reference gene. Fold changes in various *pmrC* and *naxD* transcripts in PmrA knockout and site-directed mutagenesis in relative to ATCC19606 were calculated using the 2^–△^^△^^Ct^ formula.

### 2.9 Minimal inhibitory concentration (MIC) test

Minimal inhibitory concentration (MIC) was performed using colistin sulfate and polymyxin B by employing broth microdilution in Cation-Adjusted Mueller–Hinton Broth (CAMHB), following the guidelines set by the Clinical and Laboratory Standards Institute (CLSI) (Weinstein, 2018). Each *A. baumannii* strain was diluted with LB broth to get 0.1 OD_600_ from an overnight culture, growth to 0.4 OD_600_ at 37 °C, 220 rpm till mid-log phase. The bacterial concentration was adjusted to 1 × 10^7^ CFU/mL with CAMHB. Colistin sulfate (0.5, 1, 2, 4, 6, 8, 10, 12, 16, 32, 64, 128, 256, 512 μg/mL) and polymyxin B (1, 2, 3, 4, 5, 6, 7, 8 μg/mL) was prepared at various concentrations in CAMHB. A mixture of 50 μl of *A. baumannii* and 50 μl of the polymyxin solutions was added to the wells of polystyrene 96-well microtiter plates (Corning, New York, NY, United States). The microtiter plates were then incubated overnight at 37 °C, and the OD_600_ of each well was measured. The MIC for each tested strain was determined as the lowest concentration of colistin sulfate or polymyxin B that completely inhibited the growth of the bacteria.

### 2.10 Scanning electron microscopy (SEM)

The strains were cultured on glass slides in LB medium with 2 mM IPTG for 48 h at 37°C without shake. Subsequently, they were fixed with 4% paraformaldehyde for 15 min and 2.5% glutaraldehyde overnight. The fixed specimens underwent dehydration using graded ethanol (30%, 50%, 70%, 85%, 90%, 95%, 100%), 10 min for each step, followed by critical point drying with CO_2_. Finally, the samples were coated with 15 nm diameter gold-palladium beads and photographed using a Philips XL-30 scanning electron microscope at 20 kV (Wen et al., 2017a).

## 3 Results

### 3.1 Linear map comparison, phylogeny and sequence analysis of PmrA

**FIGURE 1:**
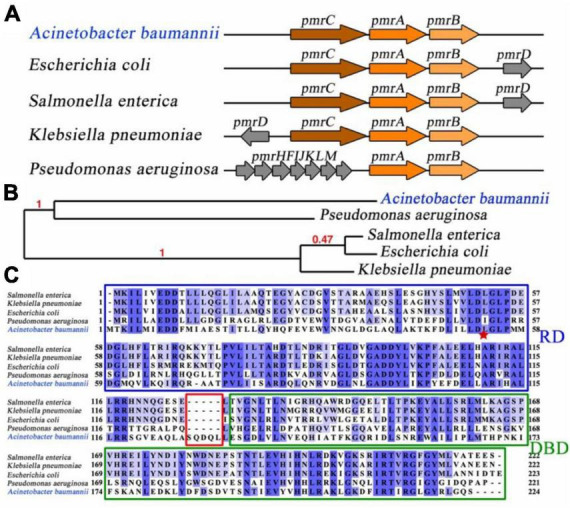
Linear map comparison, evolution, and sequence analysis of PmrA. **(A)** Linear map comparison of the *pmr* operon in different bacterial species. *pmr* operon involved PmrA, PmrB, and PmrC in *Acinetobacter baumannii*. **(B)** To determine the evolutionary relationship of PmrA across various bacterial species, the Phylogeny.fr program was utilized (Dereeper et al., 2008), employing maximum likelihood tree construction. Notably, PmrA from *Acinetobacter baumannii* exhibits a close evolutionary resemblance to *Pseudomonas aeruginosa*. The branch support values are highlighted in red, indicating the evolutionary similarity between the branches. **(C)** Sequence alignment of PmrA from five different bacterial Species. The receiver domain and DNA binding domain are represented. *Acinetobacter baumannii* PmrA shows moderate sequence identification (around 35%) compared to other bacterial species. The red star represent residues that are potential conserved phosphorylation sites.

*Pmr* operon exhibited identical core components of PmrA, PmrB, and PmrC across various bacterial species. An essential intermediary protein PmrD, can stabilizing the activated form of PmrA, plays a critical role in connecting the two-component systems in *Escherichia coli* (Wang et al., 2021), *Salmonella enterica* (Pescaretti et al., 2011) and *Klebsiella pneumonia* (Lou et al., 2015). Interestingly, the *pmrD* gene was not appear to be observed in *A. baumannii*, suggesting the existence of alternative mechanisms for coordinated regulation (Figure 1A). Comparative analysis of the *pmrA* gene from 5 distinct bacterial species revealed that *A. baumannii* PmrA has a close evolutionary affinity with *Pseudomonas aeruginosa* PmrA, as indicated by phylogeny analysis (Dereeper et al., 2008; Figure 1B).

Sequence alignment of *A. baumannii* PmrA was conducted by CLUSTALW program (Thompson et al., 1994), showed moderate sequence identity (around 35%) compared to other bacterial species. However, it is worth noting that the linker region between the RD and DBD of *A. baumannii* PmrA contains an extended sequence, namely SQDQL, which provides higher flexibility for *A. baumannii* PmrA. Residue D52 is the potential conserved phosphorylation site, which is pointed with a red star (Figure 1C).

### 3.2 Purification of PmrA and its dimerization in the presence of aspartate phosphorylation analog BeF_3_^–^

**FIGURE 2:**
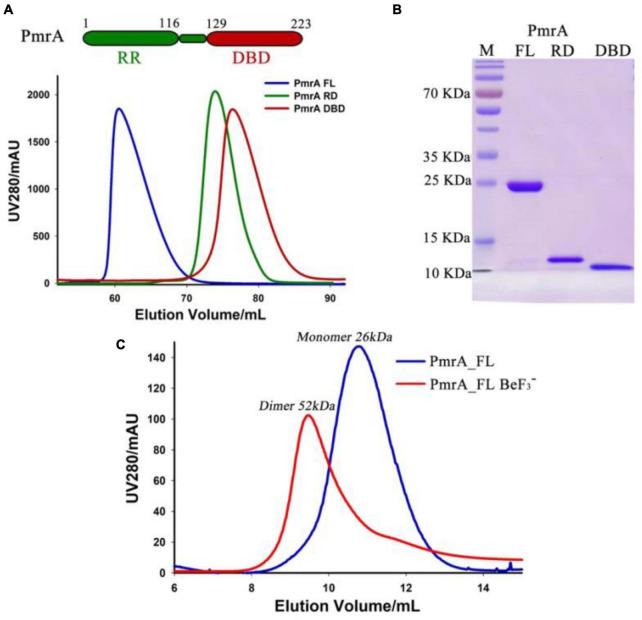
Purification of PmrA and its dimerizes in the presence of aspartate phosphorylation analog BeF_3_^–^. **(A)** The schematic view illustrates PmrA, while the size exclusion chromatography profile shows PmrA in its full-length (FL, blue), receiver domain (RD, green), and DNA binding domain (DBD, red). **(B)** Coomassie Blue Staining SDS-PAGE was performed on the SEC purified PmrA samples from panel A. **(C)** The size exclusion chromatography profile indicates that PmrA exists as a monomer but elutes as a dimer in the presence of the aspartate phosphorylation analog, BeF_3_^–^, and Mg^2+^.

The PmrA full length (FL) protein comprises of a receiver domain (RD) and DNA binding domain (DBD) belonging to the OmpR/PhoB family. To gain insight into the regulatory mechanism of the *A. baumannii* response regulator PmrA, we successfully expressed and purified the recombinant full-length PmrA (PmrA FL), as well as the PmrA receiver domain (PmrA RD) and PmrA DNA binding domain (PmrA DBD), respectively. The size exclusion chromatography (SEC) profiles indicated all constructs behaved as monomers in solution (Figure 2A). SDS-PAGE further analyzed all the purified samples from size-exclusion chromatography, followed by Coomassie Blue Staining (Figure 2B). Phosphorylation and dimerization are essential for response regulators to effectively identify target DNA and initiate downstream gene activation (Bourret, 2010; Scheller et al., 2020). Beryllium trifluoride has been extensively utilized as an aspartate phosphorylation mimic in the active state (Cho et al., 2001). In this study, full-length PmrA were incubated with BeF_3_^–^ and Mg^2+^ to mimic phosphorylation. Adding BeF_3_^–^ and Mg^2+^ induced PmrA dimerization, as indicated by size exclusion chromatography (Figure 2C).

### 3.3 Crystal structure of the PmrA receiver domain

**FIGURE 3:**
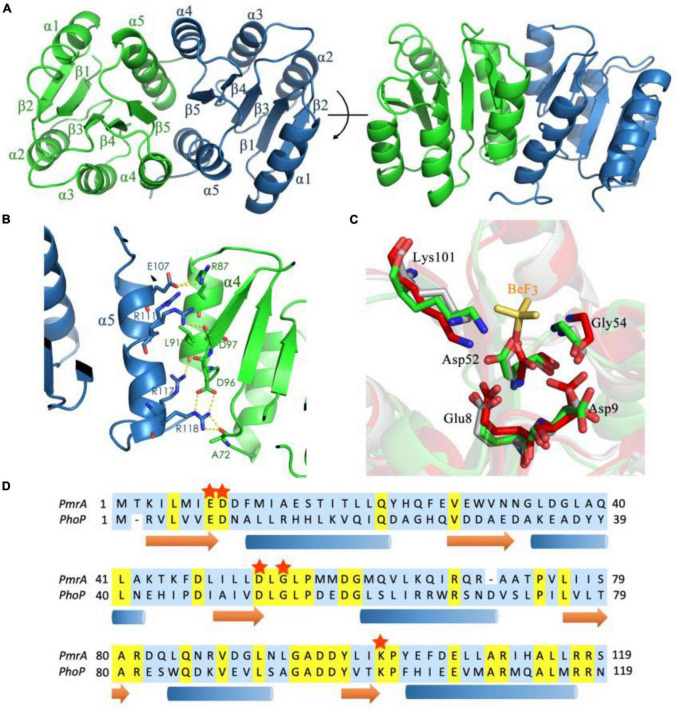
Crystal structure of the PmrA receiver domain. **(A)** The crystal structure of the PmrA receiver domain dimer is depicted in various orientations. The receiver domain of PmrA is composed of five α helices and five β strands, arranged in a parallel β-sheet configuration. The dimerization of the receiver domain occurs through the α4-β5-α5 motif. **(B)** The dimerization interface of the PmrA receiver domain is mediated by the α4-β5-α5 motif. A comprehensive interaction profile can be found in Supplementary Table 1. **(C)** Alignment of the BeF_3_^–^ binding motif of activated PhoP (PDB ID: 2PL1) (red), unactivated PhoP (PDB ID: 2PKX) (gray), and PmrA (green). The BeF_3_^–^ (yellow sticks) of PhoP is closer to Lys112 in PmrA. **(D)** The sequence alignment of the PmrA receiver domain with the extensively studied response regulator PhoP is presented. The conserved aspartate phosphorylation site and the crucial amino acids associated with it are indicated by red stars. Detailed structural statistics can be found in Table 1. The structural coordinates of the receiver domain have been deposited in the Protein Data Bank under the PDB ID: 8IMW.

The modulation of PmrA-dependent transcriptional regulation is influenced by its N-terminal receiver domain. To gain insight into the regulatory mechanism of PmrA, we conducted crystal structure analysis with a resolution of 1.6 Å. The crystal structure revealed the presence of two PmrA RD molecules in the asymmetric unit (Table 1). The receiver domain of PmrA adopts a canonical α5β5 topology, forming a central parallel β-sheet flanked by α1, α5 on one side, and α2, α3, α4 on the other side (Figure 3A). Similar to other members of the PhoB family, a significant dimerization interface consisting of α4-β5-α5 was observed, involving 9 hydrogen bonds and 13 salt bridges. This dimerization interface buries a total surface area of 999 Å, as determined by PDBPISA. Positively charged residues R111, R117, and R118 from α5 interact with negatively charged residues D97, L91, and D96 from α4 across the dimerization interface. Notably, residue R87 from α4 forms a hydrogen bond with E107 from α5 (Figure 3B). Additionally, residue R118 forms hydrogen bonds across the dimer interface with A72 in the loop between α3 and β4. Supplementary Table 1 provides a detailed list of the interactions involved in dimer formation. Structural alignment of the inactivated PmrA receiver domain with the activated and inactivated PhoP (Bachhawat and Stock, 2007) reveals a certain degree of similarity between PmrA and PhoP. The phosphorylation site of PmrA is the highly conserved aspartate residue D52 at the C-terminal end of β3. This phosphorylation process is facilitated by residues E8, D9, G54, and L101 (Figure 3C). The PmrA receiver domain shares an overall sequence identity of 26% with the well-characterized OmpR/PhoB family response regulator, PhoP (Figure 3D). Most of the residues involved in dimerization and phosphorylation, including E8, D9, D52, G54, D96, D97, R101, R111, and R118, exhibit conservation between PmrA and PhoP.

### 3.4 PmrA controls the expression of the pmrC and naxD operons

**FIGURE 4:**
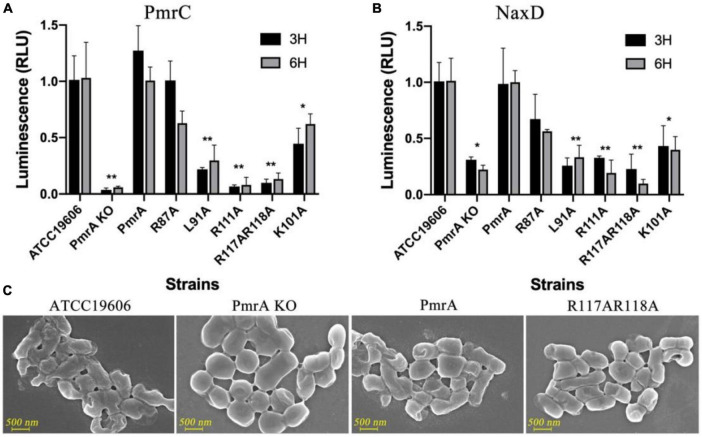
PmrA dimerization interface affected *pmrC* and *naxD* gene expression validated using real-time PCR. **(A)** The expression level of phosphoethanolamine *pmrC* gene was investigated by real-time PCR in ATCC19606, PmrA KO, PmrA, R87A, L91A, R111A, R117AR118A, and K101A strains at 3 and 6 h, respectively. Data are represented as means ± SD of 4–6 replicates. **(B)** RT-PCR examined the deacetylase NaxD gene expression level in all previously mentioned strains at 3 and 6 h. Data are represented as means ± SD of 4–6 replicates. **(C)** Field-emission scanning electron microscopy (FE-SEM) experiments of ATCC19606, PmrA KO, PmrA and, R117AR118A. Scale bars represent 500 nm. **, 0.001 < *P* < 0.01; *, 0.01 < *P* < 0.05.

A set of point mutations were introduced to the receiver domain and phosphorylation site to investigate the functional significance of these residues *in vivo*. The impact of these mutations on the transcriptional levels of *pmrC* and *naxD* was monitored, which in turn affects the modification of lipid A in *A. baumannii*. In the PmrA knockout strains, the *pmrC* and *naxD* expression were significantly reduced. The key mutations in conserved residues in PmrA dimerization, including L91, R111, R117, and R118, demonstrated an equivalent reduction to the knockout strain in *pmrC* (Figure 4A) and *naxD* transcription (Figure 4B), thereby confirming the significance of these residues in the regulatory process. Mutations in conserved residues are associated with D52 phosphorylation, including E8, D9, G54 and L101. The L101A mutations were designed to disrupt the phosphorylation of PmrA, resulting in a significant decline in the transcriptional activity of *pmrC* and *naxD*.

To further validate the changes in cell structure and membrane, we examined wildtype, PmrA knockout, PmrA complementation, and R117A R118A mutant strains using field-emission scanning electron microscopy (FE-SEM). LPS is composed of three structural components: lipid A, core oligosaccharide, and O-specific polysaccharide or O antigen. The unmodified form of lipid A can potentially alter the formation process of LPS. The findings from FE-SEM indicated the observation of rough bacterial cell surfaces in wild-type *A. baumannii* and complement strain. In contrast, the *pmrA* knockout and mutant strains of *A. baumannii* had smooth cell surfaces without lipid A modification over the cell membrane (Figure 4C).

### 3.5 Antibiotic resistance

The data obtained from minimum inhibitory concentration (MIC) has confirmed the decreased susceptibility of PmrA R87A, L91A, R117AR118A, and L101A strains to polymyxin B and colistin sulfate. The MICs of PmrA knockout strains has noticeably reduced (exceeding four-fold reduction) for polymyxin B and colistin sulfate. Although the R87A strain did not exhibit significantly changed in the expression of genes associated with lipid A modification, it did demonstrate significant alterations in the minimum inhibitory concentration (MIC) of polymyxin B and colistin sulfate. The strain L91A, R111A, and R117AR118A showed a four-fold reduction in MIC of polymyxin B and a 50-fold reduction in MIC for colistin sulfate, respectively. As anticipated, the K101A mutant displayed a potent reduction of MIC for colistin sulfate and polymyxin B, exceeding four-fold (Table 2 and Supplementary Figure 2).

**TABLE 2:** MIC values of the ATCC19606, PmrA knockout, PmrA WT, PmrA mutant strains of *A. baumannii*.

Strain	MIC (μ g/mL)	
	Colistin sulfate	Polymyxin B
ATCC19606	256	8
ΔPmrA	4	2
PmrA_WT	256	7
PmrA_R87A	4	2
PmrA_L91A	8	2
PmrA_R111A	64	6
PmrA_R117AR118A	8	2
PmrA_K101A	2	2

### 3.6 Identification of a conserved palindromic sequence as the likely PmrA binding site

**FIGURE 5:**
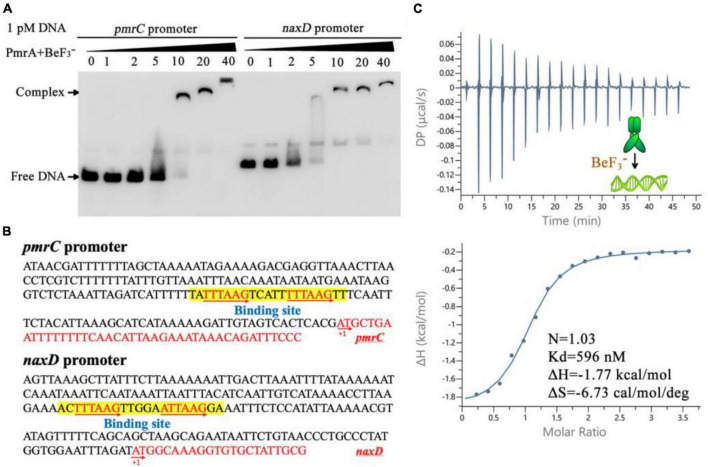
PmrA binds to *pmrC* and *naxD* promoter sequences. **(A)** Electrophoretic mobility shift assay validates that the PmrA interaction with the *pmrC* and *naxD* promoter DNA. **(B)** The schematic location of the PmrA binding boxes in the *pmrC* and *naxD* promoter, respectively. The 200+ bp nucleotide sequence upstream of the PmrC and NaxD start codon (red sequence) containing the PmrA cis-acting elements (boxed sequences). The transcription start site is indicated as +1. **(C)** The isothermal titration calorimetry of PmrA with the intercistronic DNA revealed the stoichiometry (N), dissociation constant (Kd), enthalpy (H), and entropy (S).

The modifications of Lipid A was mediated by the *pmrC* and *naxD* genes, specifically through phosphoethanolamine and deacetylase and plays a crucial role in mediating polymyxin resistance in *A. baumannii* (Chin et al., 2015; Seleim et al., 2022). Quantitative real-time PCR results have shown that expression of the *pmrC* and *naxD* operons is controlled by PmrA. To evaluate the binding ability of PmrA regulator to the *pmrC* and *naxD* promoter, a 200 bp fragment of predicted *pmrC* and *naxD* promoter regions was amplified by PCR and labeled with biotin to facilitate the investigation of its interaction with the PmrA protein by EMSA. The results demonstrated the ability of PmrA protein to bind with the specific sequence, resulting in the inhibition of motility of the *pmrC* and *naxD* promote probe, as seen by EMSA (Figure 5A). The analysis of the promoter sequences in the *pmrC* and *naxD* genes revealed highly conserved PmrA binding sites and TTTAAGNNNNNTTTAAG boxes (Figure 5B). These findings indicate a perfect 6-bp direct-repeat DNA sequence with 5 bp gap located upstream of the *pmrC* and *naxD* gene. Mutations in the TTTAAG region resulted in the absence of any interaction between the PmrA protein and the mutated promoter DNA, as evidenced by the EMSA experiment (Supplementary Figure 1A).

In order to explore the direct interaction between PmrA and the conserved sequence, a 21-base-pair DNA fragment (TATTTAAGTCATTTTTAAGTT) was synthesized, and the binding between PmrA and this DNA sequence was evaluated using isothermal titration calorimetry (ITC) (Figure 5C). The PmrA, when supplemented with BeF_3_^–^, showed a significant binding affinity with the direct-repeat DNA. The interaction between these molecules is characterized by an equilibrium dissociation constant (Kd) of 596 nM, a molar reaction enthalpy (H) of −1.77 kcal/mol, and an entropy of −6.73 cal/mol/deg (Figure 5C). Nevertheless, isothermal titration calorimetry experiment cross validated no interaction between PmrA and mutated DNA (TACCTAGCTCATTCCTAGCTT) (Supplementary Figure 1B). Additionally, in the absence of BeF_3_^–^ and magnesium, this experiment did not demonstrate any interaction between PmrA and its target DNA (Supplementary Figure 1C).

## 4 Discussion

PmrAB TCS is the critical regulator of the phosphoethanolamine transferase PmrC and deacetylase NaxD involved in polymyxin resistance of *A. baumannii*. In addition to its role in polymyxin resistance, PmrAB has been also reported to associate with decrease in biofilm formation capacity, growth rate, fitness, and virulence (Yoon et al., 2013; Farshadzadeh et al., 2018). PmrC played a crucial role in maintaining outer membrane (OM) integrity and has been shown to be linked to bacterial virulence and survival (Sinha et al., 2019). Furthermore, NaxD plays a vital role in resistance to the antimicrobial peptide polymyxin B, as well as in replication within macrophages and *in vivo* virulence (Llewellyn et al., 2012).

*Pmr* operon of some bacterial strains such as *E. coli*, *S. enterica*, and *K. pneumoniae* encompasses the PmrA, PmrB and PmrC genes and an extra gene encoding a small adapter protein called PmrD. Notably, the activity of the PmrA-PmrB system is regulated by the PmrD protein at a post-transcriptional level (Kox et al., 2000). In *A. baumannii*, the *pmr* operon share the same core components of PmrA, PmrB, and PmrC. The absence of PmrD indicates that the activation of the PmrA–PmrB system could possibly involve other proteins. In the absence of PmrD, it is conceivable that the alternative coordinated mechanisms may regulate this system. However, it is worth noting that although the core components of the *pmr* operon remain consistent across other *Acinetobacter* species, variations in sequence and evolutionary characteristics are evident in the *A. baumannii* compared to the PmrAs that exist across other bacterial species.

Sequence analysis revealed an extended sequence of PmrA in *A. baumannii*, particularly SQDQL, located between the receiver domain (RD) and the C-terminal DNA-binding domain (DBD). Similar to other members of the OmpR/PhoB family, a linker region between these two domains allows the protein to adopt different conformations, such as the “tucked” state (N- and C-terminal domains in proximity) and “extended” state (N- and C-terminal domains distal) (Palethorpe et al., 2022). Extending an additional 5-residues provides the PmrA with remarkable flexibility, which is believed to influence the phosphorylation signaling from the receiver domain (RD) to the C-terminal DNA-binding domain, playing a crucial role in facilitating proper DNA binding.

The phosphorylation simulations results indicated that similar to the classical OmpR/PhoB family response regulator, phosphorylation mediates dimerization and activation (Bachhawat and Stock, 2007). Dimerization of the full-length PmrA will take place in the existence of the aspartate phosphorylation analog BeF_3_^–^. Furthermore, we provide structural insights into the PmrA receiver domain in this study. PmrA belongs to the bacterial OmpR/PhoB response regulator family and shares secondary structure elements and a two-domain organization with other family members. PmrA features the canonical (βα)5 repeat motif. The crystal structure of PmrA reported by Palethorpe et al. (2022), exhibits the characteristic α5/β5 fold of the receiver domain, where five parallel β-sheets alternate with five amphiphilic α-helices, resulting in the conserved β1-α1-β2-α2-β3-α3-β4-α4-β5-α5 conformation, which is consistent with our study. Interestingly, the crystal structure of PmrA also reveals the presence of R118 in two alternative conformations, sharing density between the two chains.

Our structure analysis has identified several amino acids playing a crucial role in the dimerization process, including the essential amino acids R118 and A72, L91, D96, D97, R101, R111, and R117. Noteworthy, R111 amino acids have demonstrated also captured in two alternative conformations. More interestingly, our findings underscore the relevance of multiple amino acids in the phosphorylation process, such as E8, D9, D52, G54, and L101. It is necessary to emphasize that most of the residues involved in both dimerization and phosphorylation, specifically E8, D9, D52, G54, D96, D97, R101, R111, and R118, were exceptionally conserved among the OmpR/PhoB family of response regulators.

We further validated the impact of mutations in these pivotal amino acids on the expression of *pmrC* and *naxD* genes, focusing on R111 and R118. We observed an intimate relationship between PmrA mutations and the deformation of bacterial surface morphology. Thoroughly, a knockout of PmrA or a mutation of R118A could result in an abnormal smoothness of the cell surface in *A. baumannii* strains. In parallel, minimum inhibitory concentration (MIC) data revealed that both the gene knockout and the specific mutations of a specific region of the PmrA, including R87A, L91A, R117AR118A, and L101A, resulted in reduced MIC values of *A. baumannii* against colistin sulfate, and polymyxin B.

PmrA has been reported to perform a regulatory role in the expression of PmrC and NaxD gene. It has been suggested that PmrA binds to the promoter regions of these genes and potentially influences their transcription. The predicted binding site sequence is 5’-HTTAAD N5 HTTAAD (Chin et al., 2015; Palethorpe et al., 2022). In line with these studies, our results from the EMSA experiment confirmed the interaction between the PmrA and the promoter region of *pmrC* and *naxD* genes. Sequence analysis reveals a highly conserved and precise DNA binding site (a direct-repeat DNA sequence TTTAAGNNNNNTTTAAG) situated upstream of the *pmrC* and *naxD* gene, which was further validated by the results obtained from an isothermal titration calorimetry experiment. Moreover, the DNA-binding motifs of PmrA appear homologous with other bacterial species. For example, the PmrA recognition sequence of *S. enterica* was found to consist of a direct repeat (YTTAAK) (Aguirre et al., 2000), while it consists of two half-sites, 5’-CTTAAT-3’, and 5’-CCTAAG-3’, in *K. pneumoniae* (Lou et al., 2014).

Beside their crucial contribution to polymyxin tolerance, PmrA, PmrC, and NaxD are also played significant role in biofilm formation, virulence, and bacteria survival in some gram-negative bacteria such as *Escherichia coli*, *Salmonella*, *Klebsiella pneumoniae*, and *Pseudomonas aeruginosa*. Taking *Salmonella as an example*, PmrA can be activated *in vivo* through direct or indirect mechanisms, leading to the regulation of genes involved in lipopolysaccharide modification. This process contributed to the bacterium’s survival in both host and non-host environments (Gunn, 2008). In *E. coli*, *pmrAB* has been also reported to influence the cell’s resistance to antimicrobial peptides, toxic levels of iron, deoxycholate, and contributes to virulence (Warner et al., 2013).

In conclusion, our findings provide strong evidence that PmrA operates similarly to other two-domain response regulators, employing a distinct activation mechanism where phosphorylation accelerates the dimerization of the effector domain. The physiological relationship between PmrA and polymyxin tolerance was confirmed, highlighting the potential of the PmrAB TCS as a target for developing novel antibiotic agents to combat bacterial polymyxin resistance in *A. baumannii*.

## Data availability statement

The datasets presented in this study can be found in online repositories. The names of the repository/repositories and accession number(s) can be found in this article/Supplementary material.

## Author contributions

ZO: Writing – original draft, Writing – review & editing. WH: Writing – review & editing. MJ: Writing – review & editing. QY: Writing – review & editing. YG: Writing – review & editing. MR: Writing – review & editing. QQ: Writing – review & editing. JZ: Writing – review & editing. QS: Writing – review & editing. FZ: Writing – review & editing. YW: Writing – original draft, Writing – review & editing.

## Footnotes

1
https://web.expasy.org/protparam/
