# Supplementary material for: Mechanistic and biophysical characterization of polymyxin resistance response regulator PmrA in Acinetobacter baumannii
Source: Front Microbiol. 2024 Feb 27;15:1293990. doi: 10.3389/fmicb.2024.1293990 (PMC10927774; doi:10.3389/fmicb.2024.1293990)

*Supplementary Material*

# Mechanistic and biophysical characterization of polymyxin resistance response regulator PmrA in *Acinetobacter baumannii*

**Zhenlin Ouyang[1*], Wenbo He[1], Min Jiao[1], Qinyue Yu[1], Yucheng Guo[1], Moath Refat[2], Qian Qin[1], Jiaxin Zhang[1], Qindong Shi[1], Fang Zheng[2], and Yurong Wen[1*]**

[1]Center for Microbiome Research of Med-X Institute, Department of Critical Care Medicine, Shaanxi Provincial Key Laboratory of Sepsis in Critical Care Medicine, The First Affiliated Hospital, Xi'an Jiaotong University, Xi'an 710061, China

[2]The Key Laboratory of Environment and Genes Related to Disease of Ministry of Education Health Science Center, Xi'an Jiaotong University, Xi'an 710061, China.

*** Correspondence:**
Dr. Zhenlin Ouyang
Center for Microbiome Research of Med-X Institute, Department of Critical Care Medicine, Shaanxi Provincial Key Laboratory of Sepsis in Critical Care Medicine, The First Affiliated Hospital, Xi'an Jiaotong University, Xi'an 710061, China. E-mail: Zhenlin.ouyang@xjtu.edu.cn

Dr. Yurong Wen:

Center for Microbiome Research of Med-X Institute, Department of Critical Care Medicine, Shaanxi Provincial Key Laboratory of Sepsis in Critical Care Medicine, The First Affiliated Hospital, Xi'an Jiaotong University, Xi'an 710061, China. E-mail: Yurong.Wen@xjtu.edu.cn

## 1 Supplementary Tables

### 1.1 **Table S1** Interactions involved in PmrA_RD dimerization

| PmrA dimerization interface | | | | |
|---|---|---|---|---|
| Monomer A | | Monomer B | | Interaction Type |
| Residues | Atom | Atom | Residues | |
| Arg 118 | HH12 | O | Ala 72 | HB |
| Arg 118 | HH22 | O | Ala 72 | HB |
| Arg 117 | HH22 | O | Leu91 | HB |
| Arg 118 | HE | OD1 | Asp96 | HB |
| Arg 118 | HH21 | OD2 | Asp96 | HB |
| Arg 111 | HH12 | OD1 | Asp97 | HB |
| Arg87 | HH21 | OE1 | Glu107 | HB |
| Arg87 | HE | OE2 | Glu107 | HB |
| Leu91 | O | HH22 | Arg117 | HB |
| Arg118 | NE | OD1 | Asp96 | SB |
| Arg118 | NE2 | OD1 | Asp96 | SB |

| | | | | |
|---|---|---|---|---|
| Arg118 | NE | OD2 | Asp96 | SB |
| Arg 111 | NH1 | OD1 | Asp97 | SB |
| Arg 111 | NH2 | OD1 | Asp97 | SB |
| Arg87 | NE | OE1 | Glu107 | SB |
| Arg87 | NE2 | OE1 | Glu107 | SB |
| Arg87 | NE | OE2 | Glu107 | SB |
| Arg87 | NE2 | OE2 | Glu107 | SB |
| Asp96 | OD1 | NE | Arg 118 | SB |
| Glu107 | OE2 | NE | Arg87 | SB |
| Glu107 | OE2 | NE1 | Arg87 | SB |

## 1.2 **Table S2** Bacterial strains used in this study

| Strain | Genotype | Source |
|---|---|---|
| *E.coli* DH5α | F- φ80 *lac*ZΔM15 Δ(*lac*ZYA-*arg*F)U169 *rec*A1 *end*A1 *hsd*R17($r_k$-, $m_k$+) *pho*A, *sup*E44 *thi*-1 *gyr*A96 *rel*A1 λ- | Thermofisher |
| *E.coli* BL21(DE3)star | F$^-$ ompT hsd($r_B^-$ $m_B^-$) dcm gal(DE3) | Thermofisher |
| *Acinetobacter baumannii* ATCC19606 | | ATCC |
| ABpmrA | ATCC19606 ΔpmrA | This study |
| ABpmrApA | ABpmrA λpAT04pmrA | This study |
| ABpmrApAmtR87A | ABpmrA λpAT04pmrA(R87A) | This study |
| ABpmrApAmtL91A | ABpmrA λpAT04pmrA(L91A) | This study |
| ABpmrApAmtR111A | ABpmrAλpAT04pmrA(R111A) | This study |
| ABpmrApAmtR117AR118A | ABpmrA λpAT04pmrA(R117AR118A) | This study |
| ABpmrApAmtK101A | ABpmrA λpAT04pmrA(K101A) | This study |

## 1.3 **Table S3** Primers used in this study

| Primers name | Sequence (5' to 3') |
|---|---|
| PmrA construction | |
| PmrA-FL-F | CATGCCATGGGCCATCATCATCATCATCACACAAAAATCTTGATGATTGAAG |
| PmrA-FL-R | GGAATTCTTATGATTGCCCCAAACG |
| PmrA-NT-R | GGAATTCTTATTGACTCGCAAGTTGAGC |
| Gene knockout and compenent | |
| pmrA-gkUP-F | GTGGGTAGTCATGGACCTGC |
| pmrA-gkUP-R | GTTCTAGGCTCGCTTTAGTTTACATG |
| pmrA-gkDN-F | TTTAAAATTTCGGGACTTCATAAAAGTGC |
| pmrA-gkDN-R | TAATGGCTGCTAAGGCAAATATTGC |
| kan-F | CATGTAAACTAAAGCGAGCCTAGAACGTGTAGGCTGGAGCTGCTTC |
| kan-R | GCACTTTTATGAAGTCCCGAAATTTTAAAATGGGAATTAGCCATGGTCC |
| pmrA-test-F | GATGATGATCGTCCACGTTTGATTG |
| pmrA-test-R | TCATCTATTGGTTTTAAACCACGACG |
| pmrA-Op-F | CGGATAACAATTTCACACAGGAAACATGACAAAAATCTTGATGATTGAAGATG |
| pmrA-Op-R | CTTCTCTCATCCGCCAAAACAGTTATGATTGCCCCAAACGGTAGCCC |
| pAT04-Op-F | CTGTTTTGGCGGATGAGAGAAG |

| | |
|---|---|
| pAT04-Op-R | GTTTCCTGTGTGAAATTGTTATCCG |
| Site directed mutagenesis | |
| R87A-F | GATCAATTACAAAACGCGGTCGATGGTTTAAATTTGGGTGCAGATG |
| R87A-R | CAAATTTAAACCATCGACCGCGTTTTGTAATTGATCTCGAGCAGAA |
| L91A-F | CAAAACCGTGTCGATGGTGCGAATTTGGGTGCAGATGATTATTTAATTA |
| L91A-R | ATCATCTGCACCCAAATTCGCACCATCGACACGGTTTTGTAATTGATC |
| R111A-F | GTTTGATGAGTTGCTTGCCGCGATTCATGCATTACTACGCCGTAGTGGAG |
| R111A-R | GCGTAGTAATGCATGAATCGCGGCAAGCAACTCATCAAACTCATAAGG |
| R117AR118A-F | CGTATTCATGCATTACTAGCGGCGAGTGGAGTAGAAGCTCAACTTGCGAGTC |
| R117AR118A-R | GTTGAGCTTCTACTCCACTCGCCGCTAGTAATGCATGAATACGGGCAAGCAAC |
| EMSA | |
| Biotin-PCpromoter-F | CGTGAGTGACTACAATCTTTTTATG |
| PCpromoter-R | GTCTTACAAAGATAACCGTGTTGG |
| PCpromoter-muta-F | TACCTAGCTCATTCCTAGCTTTCAATTTCTACATTAAAG |
| PCpromoter-muta-R | AACCTACGAATGACCTACGTAAAAAATGATCTAATTTAG |
| Biotin-NaxD-promoter-F | GGCTAATCAAAGTGTACAG |
| NaxD-promoter-R | CTAAATTCCACCATAGGGC |
| NaxD-promoter-muta-F | ACCCTAGCTTGGACCTAGCGAAATTTCTCCATATTAAAAAC |
| NaxD-promoter-muta-R | TCCCTACGTCCAACCTACGGTTTTCTTAAGGTTTTATGAC |
| ITC | |
| PAbindingDNA-F | TATTTAAGTCATTTTTAAGTT |
| PAbindingDNA-R | AACTTAAAAATGACTTAAATA |
| PAmutatedDNA-F | TACCTAGCTCATTCCTAGCTT |
| PAmutatedDNA-R | AACGTAGGTCATTCGTAGGTA |
| RT-PCR | |
| 16S_rRNA-F | CAGCTCGTGTCGTGAGATGT |
| 16S_rRNA-R | CGTAAGGGCCATGATGACTT |
| pmrC-qPCR-F | CTCGGGTATGCCACGTGTAG |
| pmrC-qPCR-R | TCCGGTTGATTCGCCATGAT |
| naxD-qPCR-F | GGCAAAAGCTTGCCAGACAT |
| naxD-qPCR-R | TCTGGTGAGCTGGATACCGT |

## 2 Supplementary Figures

2.1 **Figure S1.** (A) EMSA experiment of mutations in the TTTAAG region resulted in the absence of any interaction between the PmrA protein and the mutated promoter DNA. (B) Isothermal titration calorimetry experiment cross validated no interaction between the PmrA protein and the mutated DNA (TACCTAGCTCATTCCTAGCTT). (C) Isothermal titration calorimetry of PmrA interaction with the direct-repeat DNA in the absence of $BeF_3^-$ and magnesium, the PmrA can't binds to the direct-repeat DNA.

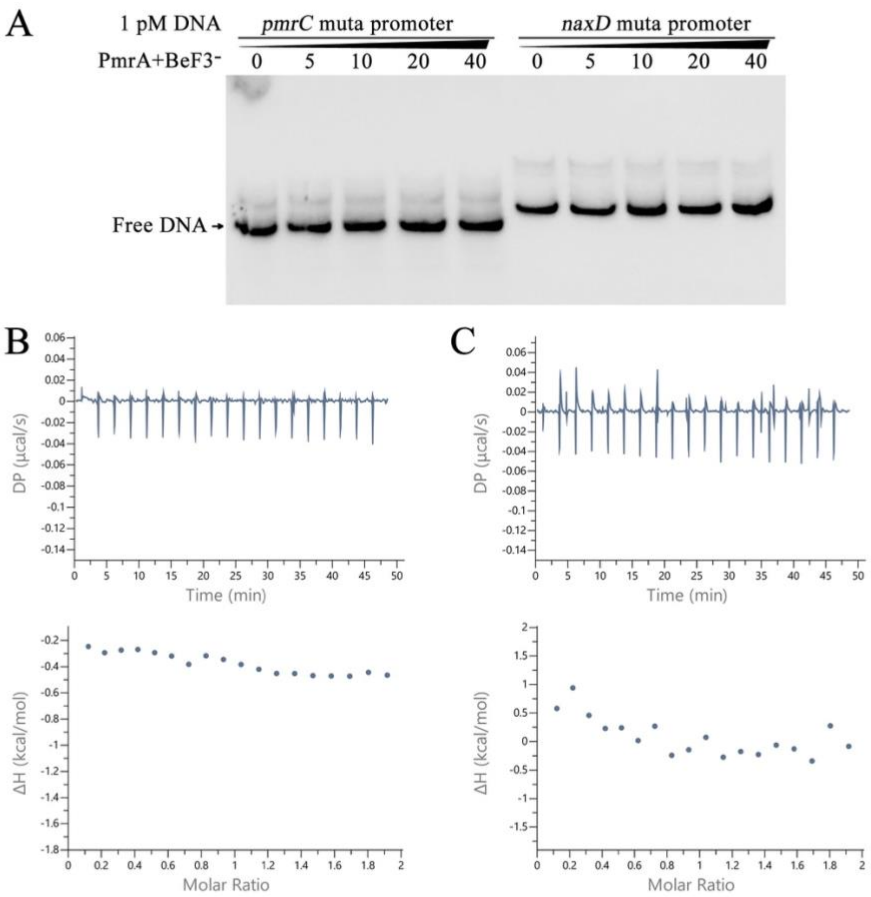


2.2 **Figure S2.** Minimal Inhibitory Concentration (MIC) of key residue mutations involved in PmrA dimerization and phosphorylation. The R87A, L91A, R117AR118A and L101A mutations significantly less susceptible to polymyxin

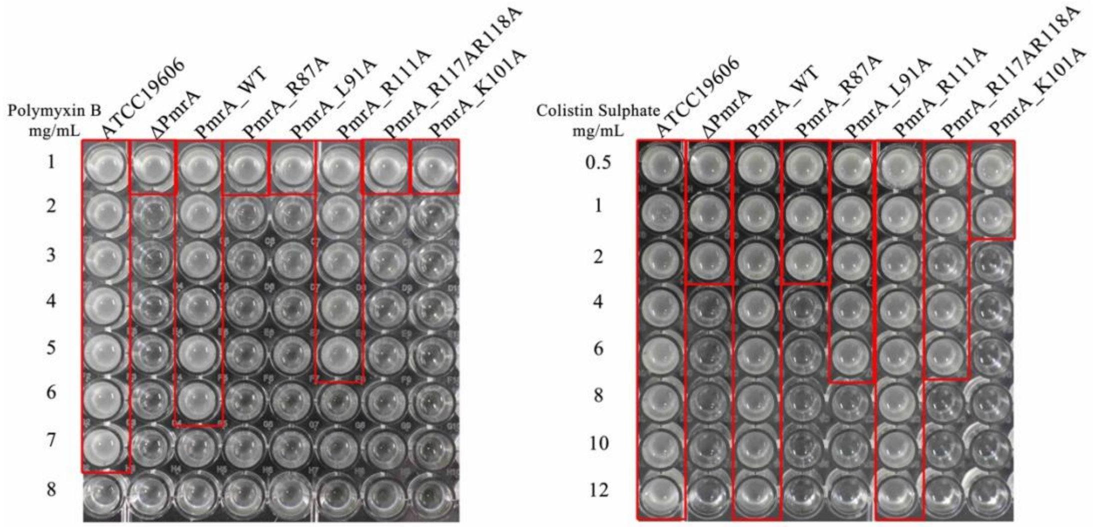

Supplement: Supplementary file 1 [file Data_Sheet_1.pdf]
